# Accurate pathologic classification of aggressive myocarditis improves insights into patient precise medicine

**DOI:** 10.1016/j.fmre.2025.09.008

**Published:** 2025-09-18

**Authors:** Xiumeng Hua, Jiayu Zhang, Han Mo, Xiao Chen, Mengni Bao, Zhe Sun, Fei Dong, Yijing Li, Shun Liu, Yiqi Zhao, Weiteng Wang, Zeyu Cui, Qian Zhao, Ningning Zhang, Jing Zhang, Xiyue Wang, Jiangping Song

**Affiliations:** a Beijing Key Laboratory of Preclinical Research and Evaluation for Cardiovascular Implant Materials, Animal Experimental Centre, Fuwai Hospital, National Center for Cardiovascular Disease, Chinese Academy of Medical Sciences and Peking Union Medical College, Beijing 100037, China; b State Key Laboratory of Cardiovascular Disease, Fuwai Hospital, National Center for Cardiovascular Diseases, Chinese Academy of Medical Sciences and Peking Union Medical College, Beijing 10037, China; c Department of Cardiovascular Surgery, Fuwai Hospital, National Center for Cardiovascular Diseases, Chinese Academy of Medical Sciences and Peking Union Medical College, Beijing 10037, China; d The Cardiomyopathy Research Group, Fuwai Hospital, National Center for Cardiovascular Diseases, Chinese Academy of Medical Sciences and Peking Union Medical College, Beijing 10037, China; e College of Biomedical Engineering, Sichuan University, Chengdu 610065, China; f Shenzhen Key Laboratory of Cardiovascular Disease, Fuwai Hospital Chinese Academy of Medical Sciences, Shenzhen 518057, China; g Department of Cardiac Surgery, Fuwai Yunnan Hospital, Chinese Academy of Medical Sciences, Affiliated Cardiovascular Hospital of Kunming Medical University, Kunming 650102, China

**Keywords:** Aggressive myocarditis, Classification, Cardiovascular pathology, Clinical feature, Deep learning, Decision tree model

## Abstract

Because the disease's heterogeneity is ignored, the current diagnosis of myocarditis with advanced heart failure is imprecise, and there are no established criteria for subtyping this type of myocarditis. We analyzed data from 83 consecutive patients with aggressive myocarditis using a deep learning method to categorize them into three prognostically relevant phenogroups (PGs) based on myocardial whole-slide images: PG1, characterized by structural damage; PG2, dominated by autoimmune response; and PG3, driven by infections. Whole-exome sequencing of all patients revealed a significant concentration of mutations in myocardial structure-related genes in PG1, with the titin gene (*TTN)* being the most frequently mutated. In contrast, mutations in immune-related genes were more prevalent in PG2 and PG3. RNA sequencing revealed unique molecular signatures: PG1 was associated with apoptosis, fibrosis, and the activation of abnormal metabolic pathways, whereas PG2 and PG3 were linked to cell damage and the activation of divergent immune pathways. Immunohistochemical staining suggested that PG1 and PG2 were characterized by T cell infiltration while PG3 was characterized by macrophage infiltration. In addition, the interleukin-6 signal transducer (IL6ST) was identified as a novel prognostic biomarker for myocarditis. A plasma IL6ST content ≥ 332.45 ng/ml was found to be associated with poor survival from recurrence symptom (hazard ratio [HR] 22.8, 95% confidence interval [CI] 2.3 to 227.2, Log-rank *p*-value < 0.0001). Finally, we used a decision tree model incorporating four clinical parameters to further validate the effectiveness of these features. This study identified three distinct PGs of aggressive myocarditis with significant differences in pathological manifestations, immunophenotyping, survival outcomes, clinical presentations, and molecular profiles. This approach improves patient selection for novel interventions and thus enables precision medicine.

## Graphical abstract

Schematic overview of the study. A myocarditis transplantation cohort was established and a digital pathology platform for myocarditis tissue was developed. An AI model was developed from pathological images to generate prognostic pathologic maps. The cohort was categorized into three subtypes, which were further analyzed based on clinical characteristics and genomics. A decision tree model was then designed to infer the subtype of each patient, providing a clinically relevant method for classification.

**Figure:**
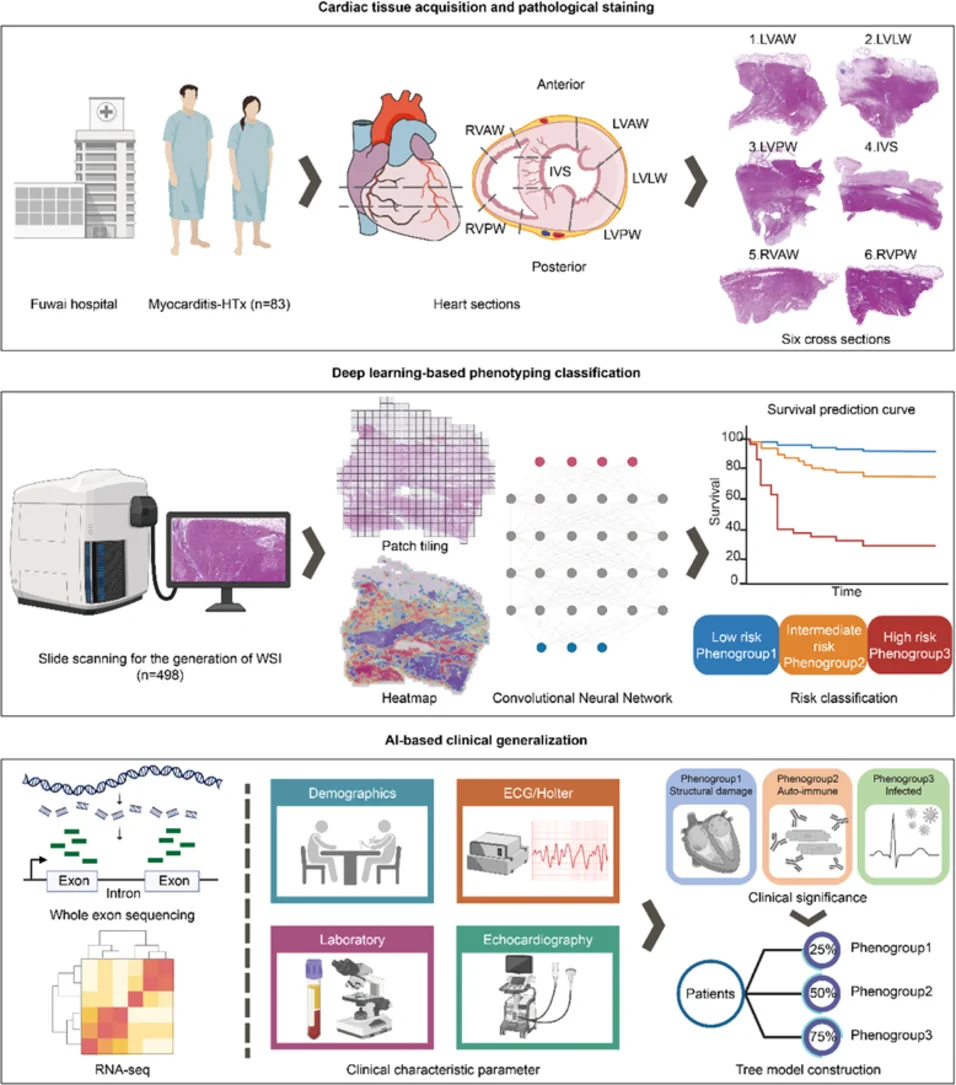

## 1 Introduction

Myocarditis is an inflammatory disease of the heart muscle caused by various etiologies, which can lead to acute heart failure, sudden death, or chronic dilated cardiomyopathy. It manifests in four types: lymphocytic myocarditis, eosinophilic myocarditis, giant cell myocarditis, and granulomatous myocarditis [1,2]. The first three types represent a heterogeneous group of patients affected by various genetic and acquired triggers. This heterogeneity makes accurate classification of myocarditis for clinical decision-making a challenge. Moreover, the prognosis varies significantly among these myocarditis types. For instance, patients with giant cell myocarditis have a median survival of 12 years, whereas most with lymphocytic myocarditis tend to recover [3,4]. Additionally, about 10 percent of these patients may progress to advanced heart failure, necessitating heart transplantation (HTx), a condition referred to as aggressive myocarditis. Traditionally, the diagnosis of myocarditis in patients with advanced heart failure has relied on measuring ejection fraction and identifying the underlying cause [5]. However, this method often lacks precision and overlooks the disease's heterogeneity. The absence of clear guidelines for classifying myocarditis subtypes further complicates accurate diagnosis and effective treatment. There is a pressing need to develop a noninvasive classification system that could more accurately capture the clinical and biological differences among patients with aggressive myocarditis, potentially guiding more targeted therapeutic approaches by grouping patients with similar pathophysiology, thereby forming more homogeneous patient populations.

The classification of myocarditis subtypes remains controversial and can be performed based on cause, histology, immunohistology, and clinicopathological and clinical criteria [1]. However, no classification system has been established for aggressive myocarditis. Cardiovascular clinicians face the challenge of utilizing the increasingly complex clinical data, including genetic profiling, advanced imaging, transcriptional data, and proteomic data, to guide patient care [6,7]. Although pathological analysis is fundamental to understanding disease, it is not yet integrated into the management and classification of cardiovascular diseases [8]. In contrast, pathological data have been used to classify tumors such as hepatocellular-cholangiocarcinoma, lung adenocarcinoma, mesothelioma, and glioma [[9], [10], [11]]. Phenomapping based upon unsupervised clustering of pathological features may help to create homogeneous myocarditis subgroups to guide the management of aggressive myocarditis patients [12].

Deep learning aids in detecting patterns between variables that explain the heterogeneity in a dataset. The development of digitized scanners allows the transformation of glass slides into whole-slide images (WSIs), which offers an opportunity for image analysis algorithms to achieve automatic and unbiased computational pathology calculations. Within the realm of cardiovascular disease, AI-based histopathology analysis plays a pivotal role in detecting, subtyping, and grading allogeneic rejection [[13], [14], [15]]. However, there remains a need for a WSI diagnostic model that can classify myocarditis in detail according to the 2013 ESC guidelines. Evidence suggests that histopathological image features in myocarditis are linked to specific pathogenic alterations, such as viral infections [[16], [17], [18]]. Besides, the 2013 ESC classification of myocarditis did not consider prognosis, which did not meet the needs of clinical management. Developing an integrated classification model directly from WSI to classify myocarditis that combines pathological, pathogenic and prognostic features is still challenging.

In this work, we proposed a cardiopathologist-level integrated phenotyping model for automatically predicting clusters and prognosis of aggressive myocarditis from annotation-free standard WSIs. Given that myocardium histopathology is the gold standard for determining the status of aggressive myocarditis, we initially classified aggressive myocarditis into prognosis-correlated groups using existing pathological samples. However, obtaining invasive histopathological samples during diagnosis can be challenging, and sometimes impossible. To facilitate non-invasive classification in future clinical practice, we further identified distinctive clinical and genomic characteristics for these prognosis-related groups. Ultimately, we determined four clinical parameters—body mass index (BMI), family history, age of onset, and proviral infection history—that effectively distinguish aggressive myocarditis.

The goal of this study is (i) to identify patterns in pathological manifestation and clinical data, cluster patients based on phenotypic similarities; (ii) to study their underlying molecular profiles and possible pathogenesis by performing whole exome sequencing (WES) and RNA-sequencing on cardiac samples of patients from each phenogroup; and (iii) to build a clinical classifier by clinical features to guide management of aggressive myocarditis.

## 2 Methods

### 2.1 Specimens and clinical data

The retrospective study involved transplant patients who were diagnosed with aggressive myocarditis. The Ethics Committee of Fuwai Hospital, Chinese Academy of Medical Sciences approved all aspects of the study, including clinical data and tissue sample collection (Approval No 2017–887). Written informed consent was obtained from all participants prior to inclusion. None of the participants received compensation. Sample collection considered the quantity and quality of tissue available for analysis and the availability of clinicopathological data. The cohort consisted of patients who underwent heart transplants at Fuwai Hospital between January 2008 and December 2021 and were confirmed to have myocarditis based on the Dallas criteria [19]. Exclusion criteria included patients with myocardial infarction, coronary heart disease, myocardial ischemia, secondary or multiple transplants, and those with implants such as ICD, CRTD, and LVAD, or other cardiomyopathy diagnoses. Finally, a total of 83 patients were included in the study. The assessed clinicopathological characteristics encompassed age, sex, family history, past infections, MRI results, cardiac ultrasound findings, electrocardiogram data, biochemical examinations, and physical examinations. The endpoint of the study was transplantation, with the initial diagnosis of clinically suspected myocarditis as the starting point [20]. Diagnostic evidence is presented in Table S1.

### 2.2 Myocarditis validation cohort

To verify the prognostic potential of plasma IL6ST, which is associated with an accelerated transplantation process, and IFNA5, linked to delayed disease progression, as biomarkers, we continuously recruited 65 untreated patients diagnosed with myocarditis at Fuwai Hospital throughout 2021. Exclusion criteria included: (1) active hepatitis B or C infection; (2) liver cirrhosis or malignant tumors; (3) pulmonary or muscle fibrosis; (4) severe systemic diseases affecting inflammatory markers; and (5) any malignant tumors. At the time of the first admission, peripheral blood was collected using an anticoagulant EDTA tube, and plasma was separated by centrifugation (4000 rpm, 4 °C, 10 min) and stored at −80 °C prior to quantitative analysis of the biomarkers. Following standardized medical treatment, a structured telephone interview was conducted to assess the primary endpoint of recurrence of myocarditis-related symptoms, which included new or aggravated chest tightness, chest pain, palpitations, or exertional dyspnea reported by the patients. During this structured interview, any new or aggravated symptoms reported would be recorded as confirmed recurrence events. Twenty patients were lost to follow-up, resulting in a total of 45 patients successfully followed up for subsequent analysis, with an average follow-up duration of 46.3 ± 8.9 months.

### 2.3 Digital pathology data collection and preprocessing

We aimed to develop a deep learning-based approach to identify myocarditis phenogroups from cardiac pathological whole slide images (WSIs). We collected H&E-stained heart sections from six areas of the heart: the left anterior ventricular wall, left lateral ventricular wall, left posterior ventricular wall, interventricular septum, right posterior ventricular wall, and right anterior ventricular wall. These samples were digitized using a Zeiss digital pathology scanner at 40 × magnification. A pathologist reviewed all WSIs along with the corresponding pathology reports, excluding any slides of poor quality, such as large debris, folds, pen marks, or blurred areas. Ultimately, we collected 498 high-quality WSIs with usable classification results from 83 patients, covering six sites per patient. In the preprocessing phase of each WSI, automated using Python 3.8.10, we eliminated blurry and uninformative background maps where more than half of the pixels had a brightness value greater than 210. We then subdivided the remaining regions into non-overlapping patches with a size of 224 × 224 pixels.

### 2.4 Deep learning model for identifying myocarditis phenogroups

We developed a deep-learning model to categorize aggressive myocarditis patients into subgroups with distinct prognoses based on histopathological images (Fig. 1). Our method integrates a powerful self-supervised foundation model for feature representation and a deep attention model for risk assessment.

**Fig. 1:**
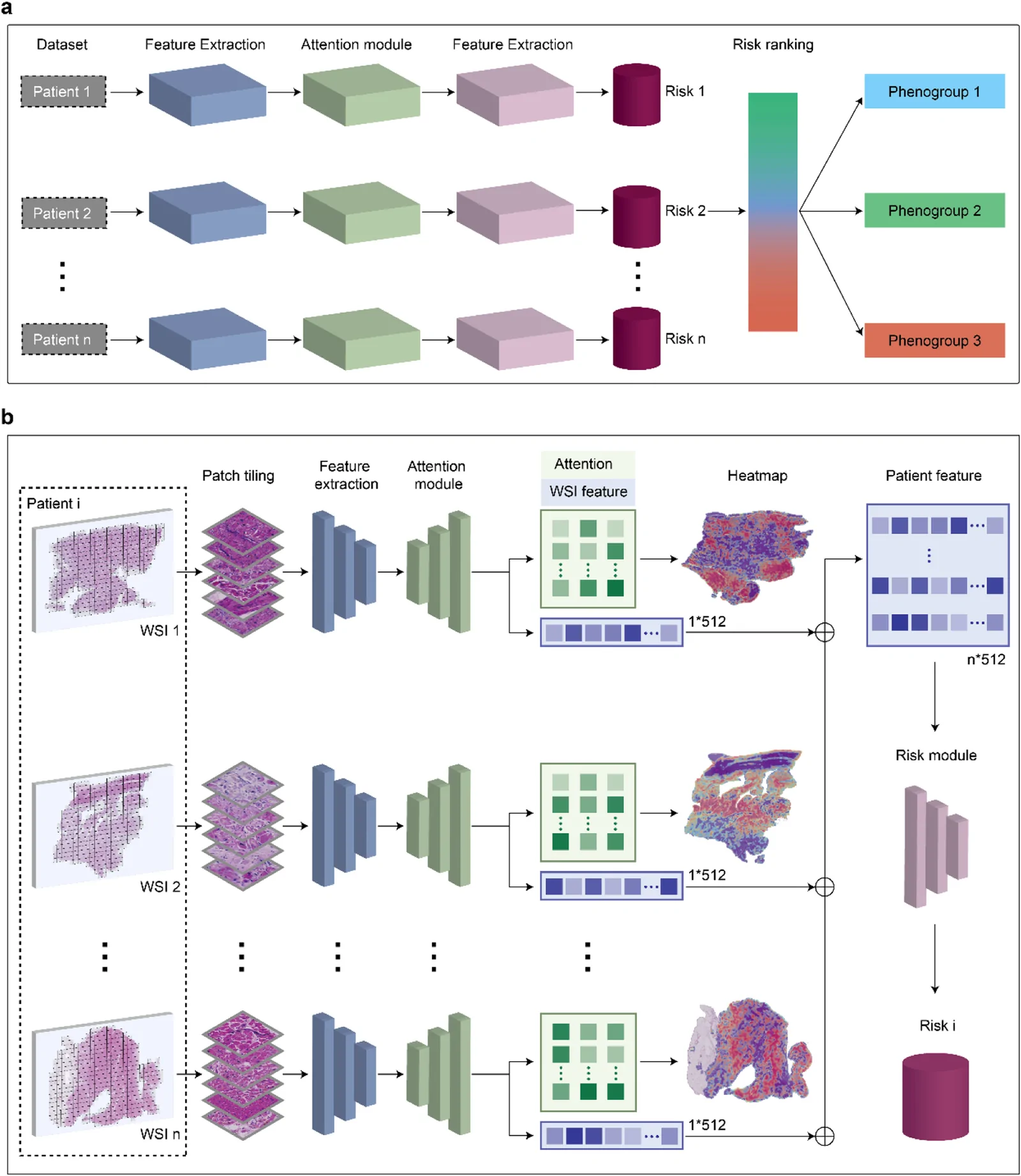
**Pipeline of prognostically relevant myocarditis classification based on pathologic WSIs.** (a) Overview of myocarditis classification through efficient pathologic image feature extraction and prediction of prognostic risk factors. The integrated patient-level features will be used to calculate risk values using the Cox model. Subsequently, by ranking these risk values from low to high, all patients can be categorized into three distinct groups. (b) Elaboration on prognostic risk prediction and exploration of pathologic appearances related to subtypes. Taking patient i containing n WSIs as an example, our process initiates by partitioning each WSI into patches. Subsequently, we extract patch-level features utilizing our foundation model. WSI-level features with a size of 1 × 512 are subsequently acquired through a feature aggregation network. The identification of predicted regions of interest involves mapping through a deep attention mechanism, aiding in the exploration of subclasses expressing relevant pathological features.

The feature extraction model (CTransPath [21]) captures features at the patch level. It is pretrained on a vast collection of unlabeled histopathological images, establishing a shared feature representation tailored for H&E-stained histopathological images. CTransPath integrates convolutional neural networks (CNN) and a multi-scale Swin Transformer architecture, enabling the capture of both local and global feature representations. Through CTransPath, each patch is transformed into a 1 × 768 dimensional feature vector. Then, these patch-level features are aggregated into WSI-level features with a dimension of 1 × 512 to predict risk scores. Note that a patient usually contains six WSIs from different heart regions: left ventricle anterior wall (LVAW), left ventricle lateral wall (LVLW), left ventricle posterior wall (LVPW), interventricular septum (IVS), right ventricle anterior wall (RVAW), and right ventricle posterior wall (RVPW). The features from all WSIs of the same patient are concatenated to compute patient-level risk parameters.

We trained our model using a five-fold cross-validation strategy and utilized the Cox proportional risk model to calculate risk values for each patient. These risk values correspond to the patient’s prognosis, with higher values indicating a poorer prognosis. Patients were categorized into three groups within each fold based on their risk values, with thresholds set at the 33rd and 67th percentiles, following an ascending order from low to high risk.

Additionally, we generated WSI-level heatmaps to highlight regions relevant to predictions. To ensure optimal image resolution, the heatmaps were created with an 80% patch overlap rate during WSI patching. Patches identified by the model as most important are labeled with warmer colors, serving as a visual reference for doctors to explore prognostically significant pathological regions.

### 2.5 DNA extraction and whole-exome sequencing (WES)

Genomic DNA was extracted from the peripheral blood of each sample, fragmented to an average size of 180∼280 bp, and DNA libraries were constructed using the established Illumina paired-end protocol. Exome capture was performed using the Agilent SureSelect Human All ExonV6 Kit (Agilent Technologies, Santa Clara, CA, USA) according to the manufacturer’s instructions. Sequencing was conducted on the Illumina Novaseq 6000 platform (Illumina Inc., San Diego, CA, USA), generating 150 bp counterpart reads. A minimum coverage of 10 × (with an average coverage of 100 ×) was achieved for approximately 99% of the genome.

### 2.6 Data analysis and SNP/indel calling

After sequencing, the bcl2fastq software (Illumina) was used for base call file conversion and demultiplexing. The resulting fastq data underwent in-house quality control to remove low-quality reads, after which they were aligned to the reference human genome (hs37d5) using the Burrows-Wheeler Aligner (BWA). Duplicate reads were marked using Sambamba tools. Single-nucleotide variants (SNVs) and indels were called using SAMTool to generate gVCF files [22]. The raw calls of SNVs and INDELs were further filtered with the following inclusion thresholds: 1) read depth > 4; 2) root-mean-square mapping quality of covering reads > 30; 3) variant quality score > 20.

### 2.7 Annotation and rare variants filtering

Variants in desmosomal genes and myocarditis-related genes (Table S3) were screened. Myocarditis-related genes were sourced from two main databases: first, a search in DisGeNET [23] using ‘myocarditis' as a keyword identified 285 genes with at least one PubMed report related to myocarditis. Additionally, three myocarditis-related gene sets were retrieved from the Gene Set Enrichment Analysis (GSEA) database using the same keyword (https://www.gsea-msigdb.org/gsea/msigdb/human/search.jsp). For a detailed gene list, please refer to Table S4. The genes from these two databases were combined to create a myocarditis-related gene panel for screening.

Annotation was performed using ANNOVAR (2019 June). The annotation included minor allele frequencies from public control datasets, as well as deleteriousness and conservation scores, enabling further filtering and assessment of the likely pathogenicity of variants. Filtering of rare variants was performed as follows: (1) variants with a minor allele frequency (MAF) < 0.01 in all 4 public population databases (1000 Genomes, ESP6500siv2_all, GnomAD_ALL and GnomAD_EAS) [24]. (2) Only single-nucleotide variants (SNVs) occurring in exons or splice sites (within 10 bp of the splicing junction) were further analyzed, as the focus was on amino acid changes. (3) Synonymous SNVs not predicted to alter amino acids by dbscSNV, and small non-frameshift indels (< 10 bp) in regions defined as repeats by RepeatMasker, were discarded. (4) Variations were further screened based on scores from SIFT, Polyphen, MutationTaster, and CADD software. Potentially deleterious variants were retained if more than half of these tools indicated a harmful effect. Variants that did not affect alternative splicing (in cases involving > 2 bp) were removed.

To more accurately predict the harmfulness of variations, the classification system of the American College of Medical Genetics and Genomics (ACMG) [25] was used. Variations were classified into five categories: pathogenic (P), likely pathogenic (LP), uncertain significance (VUS), likely benign, and benign.

### 2.8 Construction and analysis of transcriptome sequencing database

The construction of a transcriptome sequencing database involves several key steps: (1) Total RNA extraction is performed from a tissue sample, with RNA purity, concentration, and integrity (RIN value) being carefully assessed. (2) mRNA enrichment and fragmentation are achieved by using magnetic beads with Oligo (dT) to enrich eukaryotic mRNA, followed by fragmentation using specific reagents. (3) cDNA synthesis begins with the synthesis of the first cDNA strand using a random hexamer primer and reverse transcriptase, and the second strand is synthesized by adding buffer, dNTPs, RNase H, and DNA polymerase I, with double-stranded cDNA then purified. (4) End repair and adapter ligation are performed on the double-stranded cDNA, preparing for (5) PCR enrichment, where the cDNA library is amplified and quality-checked. The final step (6) high-throughput sequencing involves loading the prepared library onto a sequencer.

The analysis of sequencing data includes: (1) Sequence alignment using tools HISAT2 to align reads to a reference genome or transcriptome; (2) Quantification of gene expression levels with software featureCounts; (3) Differential expression analysis to identify significant changes in gene expression under different conditions, using tool edgeR; (4) Functional enrichment analysis with databases Gene Ontology (GO) and Kyoto Encyclopedia of Genes and Genomes (KEGG) elucidates the biological significance of differentially expressed genes; (5) Visualization of the results is done using tools R, generating heat maps, scatter maps, and volcano maps to effectively show the findings.

### 2.9 GSEA, gene set variation analysis (GSVA) and single-sample GSEA (ssGSEA)

GSEA was performed to explore enriched pathways and interpret RNA-seq data using predefined gene sets from the Molecular Signatures Database (v.7.1) in GSEA software (v4.0) [26,27]. All basic and advanced settings were kept at their default values. In the subsection on digital pathology data collection and preprocessing, single-sample GSEA scores were calculated for each sample using the “gsva” function in the R package ‘GSVA’ v1.42 [28]. The gene sets used for enrichment analysis included the KEGG pathway-related gene set C2, the GO term-related gene set C5, and the immune-related gene set C7 (All obtained from http://www.gsea-msigdb.org/gsea/msigdb). Additionally, ssGSEA was employed to quantify immune cell infiltration levels using a predefined panel of immune cell marker genes (https://ckg.readthedocs.io/en/latest/notebooks/recipes/ssGSEA_with_PCA.html). The ssGSEA enrichment scores for each immune cell type were calculated, and mean scores across phenogroups were visualized via hierarchical clustering using the “pheatmap” package (v1.0.12), with row-scaled z-scores illustrating differential immunoinfiltration patterns among PG subtypes.

### 2.10 Immunohistochemical analysis and quantitative enzyme-linked immunosorbent assay (ELISA)

Immunohistochemical staining for CD3, CD68, and MPO was conducted following a standardized protocol. Formalin-fixed paraffin-embedded sections of cardiac tissue were dewaxed using methanol, subjected to antigen retrieval via heat mediation in an EDTA buffer (pH 9.0), and blocked for 30 min. Sections were then incubated overnight at 4 °C with anti-CD3 antibody (Proteintech, 17,617–1-AP), anti-CD68 antibody (Proteintech, 66,231–2-Ig), or anti-MPO antibody (Proteintech, 66,177–1-Ig). Subsequently, a secondary antibody, HRP-conjugated rabbit or mouse IgG, was applied at room temperature. The entire slice was scanned using a digital scanner, and the number of CD3, CD4, and MPO-positive cells within the slice was quantified using Image-Pro Plus Version 6.0 (Media Cybernetics). Frozen plasma samples stored at −80 °C were rapidly thawed at 37 °C and then placed on ice. Human IFN-α5 ELISA KIT (JM-6604H1, JINGMEI) and human gp130 (IL6ST) ELISA KIT (CSB-E04571h-IS, CUSABIO) were utilized for the analysis of plasma samples. All procedures were performed in accordance with the manufacturer's protocol.

### 2.11 Construction and internal validation of the decision tree model

To translate the deep learning-derived PGs into a clinically applicable tool, we constructed a decision tree classifier using readily available clinical features. Based on prior statistical analysis demonstrating significant differences across the three PGs (Table 1), we selected BMI, presence of a familial history of myocarditis/cardiomyopathy, age at symptom onset, and history of proviral infection within one year prior to cardiac symptoms as predictors, with pathological phenogroup assignment as the dependent variable. We employed the rpart() function within the R package "rpart" to build the model. To rigorously evaluate performance and mitigate overfitting, we implemented a robust internal validation strategy: the entire cohort of 83 patients was randomly partitioned into a training set (70%, *n* = 58) and a hold-out test set (30%, *n* = 25). The model was trained exclusively on the training set, with model complexity controlled by adjusting parameters (minsplit, minbucket) and selecting the optimal complexity parameter via 10-fold cross-validation within the training set. The predictive accuracy of the final optimized model was then evaluated on the independent test set. To account for variability in random splitting, this entire process (70:30 split, training with internal CV, testing) was repeated 10 times, and the reported classification accuracy of 70% represents the average accuracy across these 10 independent test sets. The final decision tree structure was visualized using the rpart.plot package.

**Table 1:** . **Clinical characteristics stratified by pathological phenogroup.**

Clinical characteristic	Phenogroup 1 (*n* = 28)	Phenogroup 2 (*n* = 25)	Phenogroup 3 (*n* = 30)	*p-* Value
Age of onset, years	30.79 ± 14.11	38.24 ± 14.12	39.57 ± 14.42	***0.049***
Male, n (%)	21 (75)	15 (60)	15 (50)	0.146
Familial disease, n (%)	9 (32)	1 (4)	7 (23)	***0.036***
Smoking, n (%)	6 (21)	8 (32)	9 (30)	0.651
Drinking, n (%)	8 (29)	8 (32)	13 (43)	0.467
BMI, kg/m²	21.96 ± 2.74	21.93 ± 3.31	19.96 ± 3.25	***0.042***
Heart rate, b.p.m	75.61 ± 18.67	82.84 ± 16.13	83.48 ± 14.05	0.144
Systolic blood pressure, mm Hg	96.54 ± 10.33	101.24 ± 10.89	95.93 ± 12.72	0.189
Diastolic blood pressure, mm Hg	67.64 ± 7.23	71.54 ± 6.95	65.83 ± 7.24	***0.017***
Strenuous exercise, n (%)	9 (32)	5 (20)	10 (33)	0.498
Proviral infection, n (%)	3 (11)	7 (28)	13 (43)	***0.021***
Allergic sign, n (%)	2 (7)	3 (12)	5 (17)	0.538
Auto-immune disease, n (%)	2 (7)	3 (12)	3 (10)	0.833
NYHA functional class dayudengy ≥ III, n (%)	27 (96)	22 (88)	29 (97)	0.646
Laboratory data				
“NT-proBNP, fmol/ml	4201.09 ± 3226.45	3928.29 ± 4349.02	5595.70 ± 4813.60	0.280
“cTnI, ng/ml	0.40 ± 0.87	0.17 ± 0.21	0.26 ± 0.81	0.523
“Creatinine, mmol/L	90.58 ± 21.12	93.77 ± 23.49	106.01 ± 41.32	0.134
High-sensitivity C-reactive protein (mg/L)	3.80 ± 3.94	3.36 ± 3.44	4.45 ± 4.19	0.573
BUN, mmol/L	6.93 ± 2.50	7.74 ± 3.40	9.52 ± 3.56	***0.009***
Total protein, g/L	66.22 ± 6.75	70.82 ± 7.87	66.01 ± 6.82	***0.026***
Albumin, g/L	42.43 ± 5.19	44.90± 3.76	41.69 ± 3.51	***0.018***
Sodium, mmol/L	139.40 ± 4.14	140.66 ± 2.17	141.94 ± 1.86	***0.006***
ASAT, IU/L	34.75 ± 34.91	29.36 ± 12.77	94.79 ± 245.22	0.190
ALAT, IU/L	97.64 ± 208.91	38.05 ± 26.33	129.35 ± 361.16	0.400
ECG/Holter, n (%)				
Atrial fibrillation	8 (29)	8 (32)	5 (17)	0.380
Premature ventricular contraction	14 (50)	11 (44)	23 (77)	***0.030***
Non-sustained VT	13 (46)	9 (36)	18 (60)	0.202
Atrioventricular block	5 (18)	8 (32)	8 (27)	0.486
Cardiogenic syncope	7 (25)	3 (12)	7 (23)	0.448
Therapeutic regimen, n (%)				
ACEI/ARB	22 (79)	16 (64)	17 (57)	0.203
β-blocker	22 (79)	17 (68)	22 (73)	0.684
Spirolactone	26 (93)	23 (92)	27 (90)	0.922
Benzbromarone/allopurinol	4 (14)	5 (20)	4 (13)	0.771
Diuretic	28 (100)	24 (96)	25 (83)	***0.038***
Cardiac ultrasound				
LVEDD, mm	62.14 ± 9.83	61.125 ± 10.62	59.69 ± 12.71	0.708
LAD, mm	47.46 ± 6.08	47.2 ± 5.48	46.57 ± 7.63	0.870
LVPWT, mm	8.59 ± 1.82	8.24 ± 2.51	8.64 ± 1.85	0.766
IVST, mm	8.88 ± 2.96	7.74 ± 2.33	7.92 ± 1.02	0.172
RVEDD, mm	28.74 ± 9.65	25.88 ± 5.76	29.59 ± 9.34	0.277
LVEF,%	26.86 ± 8.13	33.61 ± 11.15	32.86 ± 10.80	***0.031***
Gene mutation				
Desmosome sarcomere related gene panel, n (%)	15 (54)	6 (24)	6 (20)	***0.013***
Myocarditis related gene panel, n (%)	6 (21)	14 (56)	12 (40)	***0.035***

NYHA, New York Heart Association; BUN, Blood Urea Nitrogen; ASAT, Aspartate Transaminase; ALAT, Alanine Transaminase; ACEI, Angiotensin-Converting Enzyme Inhibitors; ARB, Angiotensin Receptor Blockers; LVEDD, Left Ventricular End Diastolic Dimension; LAD, Left Atrial Diameter; LVPWT, Left Ventricular Posterior Wall Thickness; IVST, Interventricular Septum Thickness; RVEDD, Right Ventricular End Diastolic Dimension; LVEF, Left Ventricular Ejection Fraction. Proviral infection: The presence of a cold or fever and an increase in the percentage of lymphocytes on a routine blood test within 1 year prior to the onset of cardiac symptoms.

### 2.12 Statistical analysis

Continuous variables were presented as mean ± standard deviation, while categorical variables were expressed as frequency (percentage). The comparison among the three phenogroups was conducted using one-way ANOVA, followed by Bonferroni post-hoc testing for normally distributed data. For immunohistochemical quantitative data, the non-parametric Kruskal-Wallis test with Benjamini-Krieger-Yekutieli's two-stage step-up procedure (FDR correction) was applied for multiple comparisons. Spearman correlation analysis (for non-normal distributions) or Pearson correlation analysis (for normal distributions) was employed to evaluate associations between pathological phenogroups and clinical parameters. Receiver operating characteristic (ROC) curve analysis was performed to determine the optimal cutoff values of biomarkers (IL6ST and IFNA5) for predicting symptom recurrence, with the area under the curve (AUC) calculated using DeLong's method. Multiple linear regression analysis, adjusted for age, sex, and body mass index, identified independent risk indicators for heart failure progression. The predictive value of symptom recurrence in aggressive myocarditis was assessed using Kaplan-Meier (KM) survival curves, with hazard ratios (HR) calculated via Cox proportional-hazards regression, and 95% confidence intervals reported. All statistical analyses were performed using SPSS 19.0 (IBM, USA) with two-tailed tests; *p* < 0.05 was considered statistically significant. Graphical outputs were generated using GraphPad Prism 5.0 (GraphPad Software, USA).

## 3 Results

### 3.1 Overview and patient characteristics

The dataset for this study included 83 consecutive patients with aggressive myocarditis from Fuwai Hospital who had undergone heart transplantation. The selection pipeline is illustrated in Graphical abstract. First, cardiac WSIs were obtained from patients with aggressive myocarditis, covering six specific locations as previously reported [29]. Next, deep learning-based pathology features were generated to classify aggressive myocarditis patients into different phenotypic groups. Finally, RNA-seq and WES data were used to elucidate the underlying mechanisms underlying the different phenotypes.

The mean age of onset of the patients was 36.2 ± 14.59 years, with 61.4% of these patients being male (*n* = 51). Additionally, 27.7% of patients had a documented history of infection prior to the onset of myocarditis, and more than half of the patients presented with ventricular arrhythmias, including premature ventricular contractions and ventricular tachycardia. The clinical characteristics of the cohort, along with integrated pathologic diagnoses, are summarized in Table S2.

### 3.2 A novel pathological classification of aggressive myocarditis and comparison of clinical characteristics among phenogroups

Our weakly-supervised deep learning model classified 83 patients with aggressive myocarditis into three stable phenogroups, each associated with different clinical characteristics (Table 1). Phenogroup 1 (PG1; *n* = 28; 34%) had the youngest age of onset (30.79 ± 14.11; *p* = 0.049) and the highest proportion of family history of myocarditis (*n* = 9, 32%; *p* = 0.036). Furthermore, this group exhibited the lowest left ventricular ejection fraction (LVEF, 26.86% ± 8.13%, *p* = 0.031), with all individuals receiving diuretic treatment (*n* = 28, 100%; *p* = 0.038). Phenogroup 2 (PG2; *n* = 25; 30%) presented the highest diastolic blood pressure (71.54 ± 6.95 mmHg; *p* = 0.017), the highest total protein levels (70.82 ± 7.87 g/L; *p* = 0.026), the highest albumin levels (44.90 ± 3.76 g/L; *p* = 0.018), and the highest LVEF (33.61% ± 11.15%; *p* = 0.031). Phenogroup 3 (PG3; *n* = 30; 36%) had the highest rates of proviral infection (*n* = 13, 43%, *p* = 0.021), the lowest body mass index (BMI, 19.96 ± 3.25 kg/m², *p* = 0.018), and the highest incidence of premature ventricular contraction (*n* = 23, 77%; *p* = 0.030), along with poor renal function (BUN: 9.52 ± 3.56 mmol/L, *p* = 0.009).

### 3.3 Association of phenotypic groups with different outcomes

Given that our deep learning model classified patients into three phenogroups with distinct prognoses, we examined the survival differences across these groups (Fig. 2). Survival time was defined as the duration from the onset of symptoms to HTx. Among the phenogroups, PG3 has the shortest survival time from symptom onset to HTx, while PG1 exhibited the longest survival time (Median survival time: PG1, 8.1 years; PG2, 5.3 years; PG3, 2.02 years, *p* < 0.001). PG1 and PG2 had similar early survival rates (Gehan-Breslow-Wilcoxon test, *p* = 0.1414), but PG1 showed better late survival (Log-Rank test, *p* = 0.0409, Fig. 2a).

**Fig. 2:**
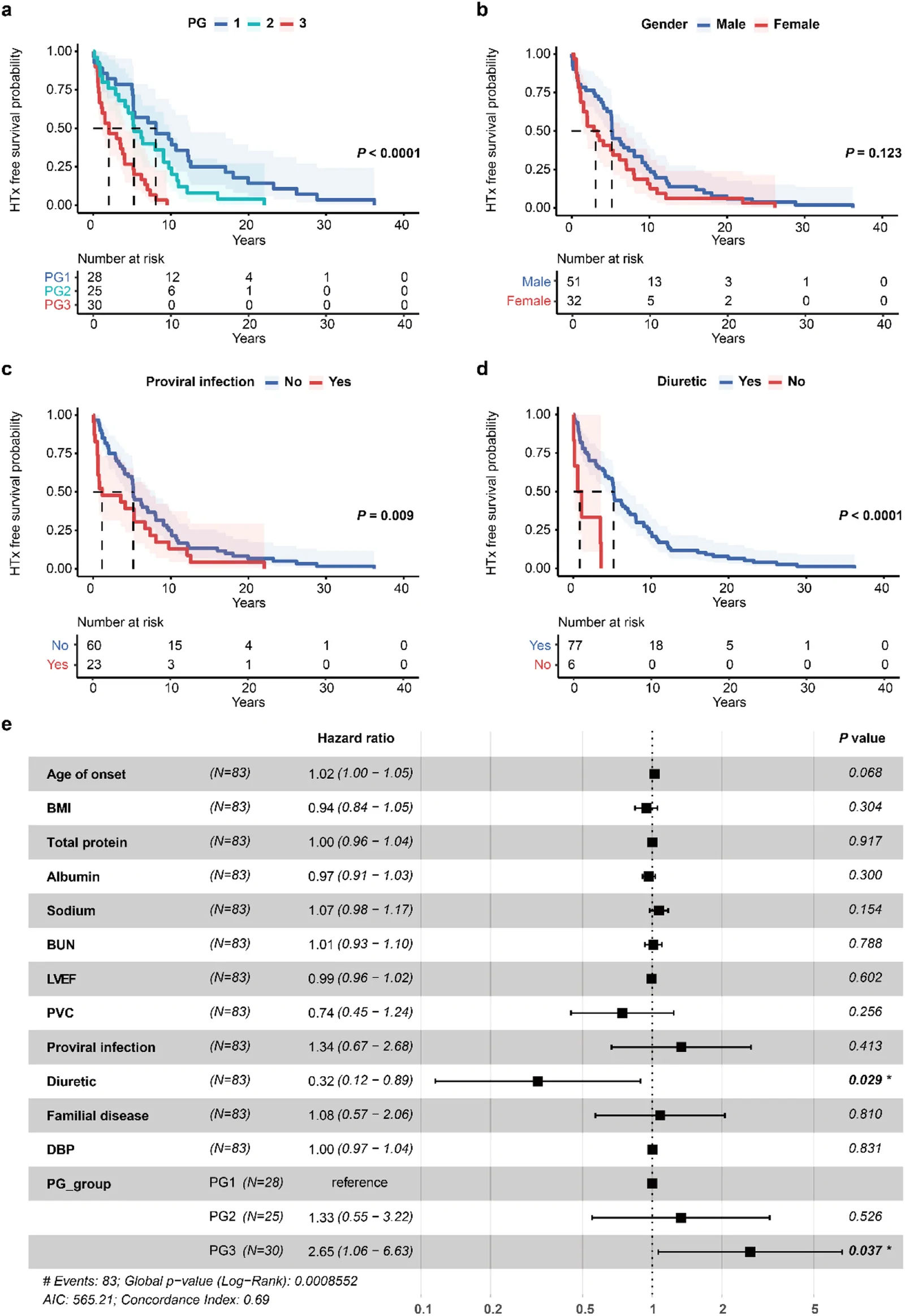
**Heart transplantation free survival stratified by phenogroup.** (a) K-M survival curve of heart transplantation in different pathological groups. (b) K-M survival curve of heart transplantation in different genders. (c) K-M survival curve of heart transplantation with or without proviral infection. (d) K-M survival curve of heart transplantation with or without diuretics. (e) Risk forest map of clinical prognostic related indicators by Cox analysis.

Previous reports have suggested that sex plays a role in the outcome of acute myocarditis, suggesting a higher risk of death or heart transplantation in women [30]. However, in our study, female patients with aggressive myocarditis had a similar risk of heart transplantation compared to male patients (Median survival: Male, 5.2 years; Female, 3.1 years, *p* = 0.1225, Fig. 2b). Viral infection is known to be the most common etiology of myocarditis [31], and the presence of viral genomes has been identified as a univariate predictor of poor prognosis [32]. Consistent with these findings, a history of proviral infection significantly impacted patient prognosis, particularly in the early stages (Median survival: proviral infection history positive, 1.15 years; proviral infection history negative, 5.18 years; *p* < 0.01, Fig. 2c). Additionally, we found that patients with myocarditis who received oral diuretic therapy had better survival (Median survival: oral diuretic therapy, 5.18 years; without oral diuretic therapy, 0.82 years, *p* < 0.001, Fig. 2d).

In addition, due to the different clinical characteristics in different phenogroups, we performed the multivariate Cox regression to identify which features were independent predictors of HTx-free survival. The clinical characteristics of patients in the three PGs were statistically different in age of onset, serum sodium level, history of proviral infection, oral diuretic treatment and so on, which were included in the multivariate Cox regression model. After multivariate Cox regression analysis, oral diuretic therapy (HR = 0.32, *p* = 0.029) and PG group (PG2 vs PG1: HR = 1.33, *p* = 0.526; PG3 vs PG1: HR = 2.65, *p* = 0.037) remained significant in predicting transplant risk (Fig. 2e).

### 3.4 Extraction of extremal tiles associated with prognosis to identify regions of interest

Another important aspect of the pathologic classification process was its interpretability. The attention scoring module we employed allowed us to assign scores to each tile for a given patient, with higher values indicating a poor prognosis and lower values indicating a favorable prognosis (Fig. 3a). This scoring system can serve as a valuable tool for pathologists to identify specific areas of interest that are relevant to the survival of each patient.

**Fig. 3:**
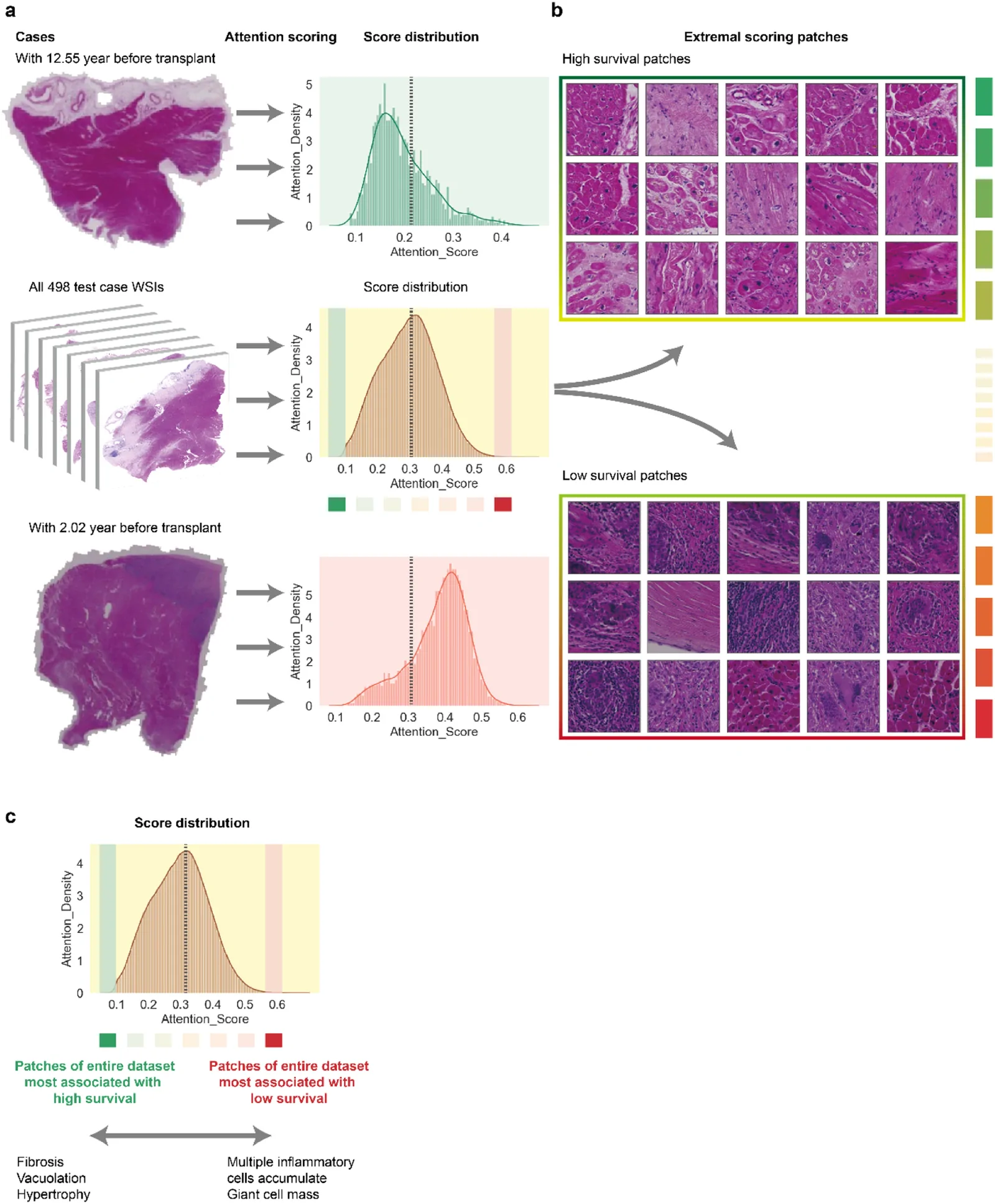
**Extraction of extremal tiles associated with prognosis to identify regions of interest.** (a) Attention scores were extracted from a total of 498 cases of whole slide images (WSI), with patients displaying fast transplant progression at the bottom and those with slow transplant progression at the top. (b) Extreme tiles related to overall fast and slow transplant progression in all patients with myocarditis were extracted and reviewed by pathologists. (c) Histological analysis revealed that tiles with high attention scores and rapid graft progression exhibited inflammatory cell aggregation and giant cell mass, while tiles with low attention scores and slow graft progression showed fibrosis, vacuolation, and disordered heart arrangement.

We aggregated scores from all 498 slides into a single distribution and then isolated those associated with high and low survival rates (Fig. 3b). Two expert pathologists independently examined these extreme tiles and found that patches associated with low survival were predominantly located in the left ventricle of the myocardium and within the myocardium layer, indicating the infiltration of immune cells and giant cell masses (Fig. 3c). In contrast, tiles associated with high survival showed higher levels of fibrosis, instances of cardiac hypertrophy, and disorganized arrangement of cardiomyocytes (Fig. 3c).

### 3.5 WES of cardiac samples to unravel genetic susceptibility of myocarditis

In order to investigate the pathogenesis of three phenogroups, WES was performed on 83 patients diagnosed with aggressive myocarditis, which revealed that 54 individuals carried variants classified as pathogenic (P), likely pathogenic (LP), or variants of uncertain significance (VUS) (Table S5). This cohort included 18 frameshift deletion mutations, 36 non-synonymous single SNVs, 5 splice site mutations, 1 start loss mutation, and 10 stop gain mutations. Additionally, 13 individuals were found to possess more than one mutation. Notably, mutations in *TTN* (10/83, 12%) and *CCR5* (4/83, 5%) were observed with relatively high frequencies.

We analyzed the mutations present in each phenogroup. PG1 predominantly harbored desmosome-related mutations (53.6%), while PG2 and PG3 primarily contained mutations associated with myocarditis (40.0% and 36.7%, respectively, Fig. 4a). Furthermore, we visualized the differential clinical features and mutation characteristics of all patients (Fig. 4b). This suggests that PG1 patients may develop cardiac inflammation as a consequence of structural issues with the heart muscle, which aligns with reports indicating that genetic variants in cardiomyopathy-associated genes are prevalent in a significant proportion of patients with acute myocarditis. Moreover, myocarditis-related gene mutations were more common in PG2 and PG3 compared to PG1 (Table 1), indicating a higher likelihood of immune-related susceptibility to myocarditis in these groups.

**Fig. 4:**
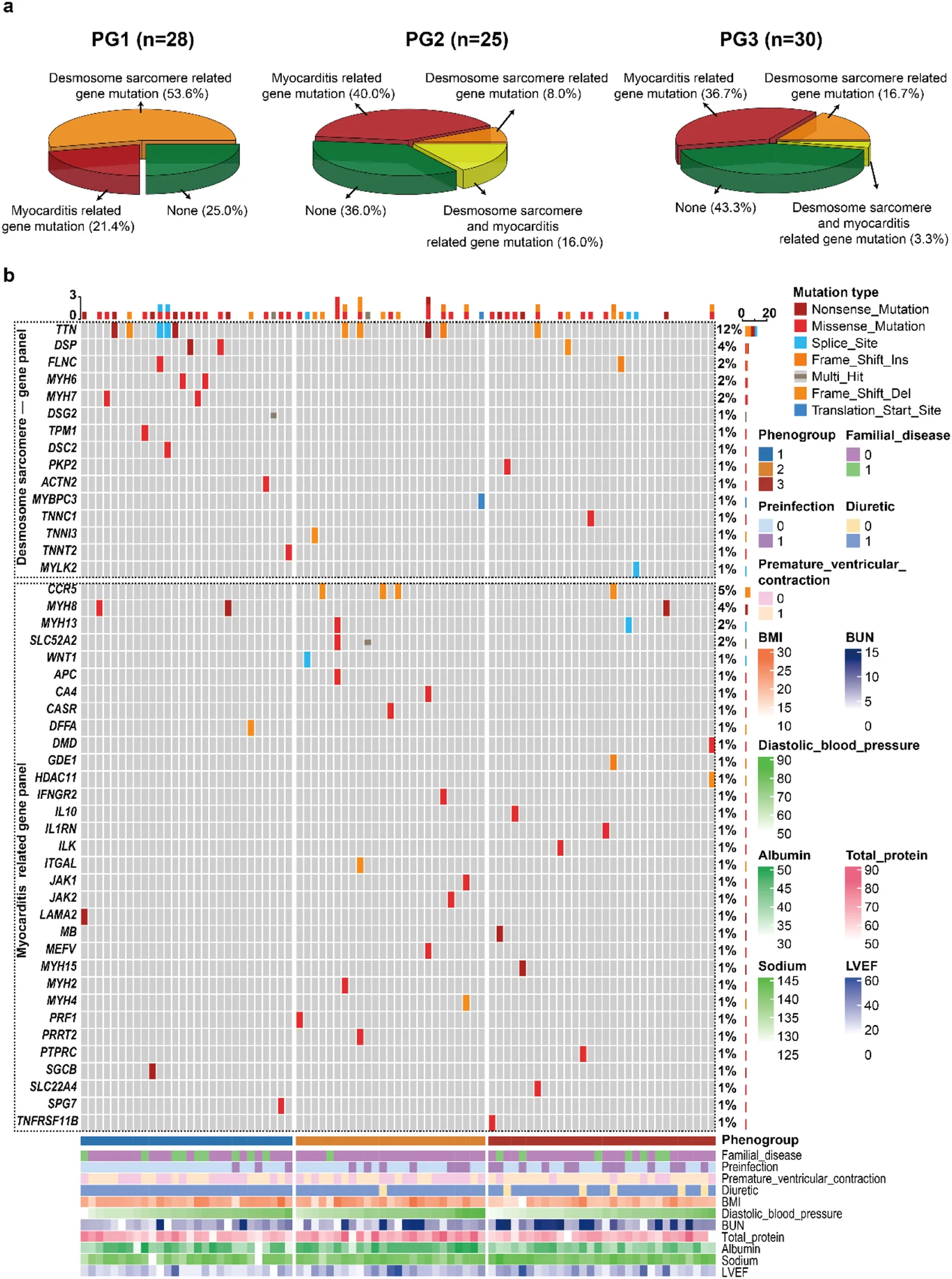
**Genetic mutation oncoplot and differential clinical indicators of three phenogroups were enriched.** (a) Displays of the proportion of mutations present in each of the three PG groups. (b) A oncoplot map that visualizes all P/LP/VUS mutations. Additionally, it includes a heat map depicting clinical characteristics that exhibit significant differences among the three groups.

### 3.6 RNA sequencing of cardiac samples to unravel underlying pathophysiology

To further understand the cardiac pathophysiological differences between the phenogroups, we conducted genome-wide transcriptome analysis through RNA sequencing of cardiac samples from a sub-cohort of 18 patients, with an equal distribution of PG 1–3 (6 patients per group) (Fig. 5a). The three groups were randomly selected, and the K-M survival curves were plotted after selection (Fig. S1). Subsequently, we performed differential expression analysis for the three pathological subtypes (Fig. S2a-c) and conducted an intersection analysis of the up-regulated and down-regulated genes (Fig. 5b and S2d). Further enrichment analysis of specific up-regulated and down-regulated genes for each subtype was carried out separately (Fig. S3 and S4), revealing significant differences between PG3 and PG1, primarily in myocardial and inflammatory pathways, with PG2 showing intermediate characteristics. GSEA enrichment analysis highlighted inflammation and abnormal activation of myocardial structure as key differences between PG3 and PG1 (Fig. 5c and S5). Furthermore, abnormal activation of ion-related pathways in PG3 was linked to frequent clinical arrhythmias. Notably, PG3 and PG2 exhibited distinct modes of inflammation activation. Additionally, GSVA revealed that PG1 displayed features associated with apoptosis, fibrosis production, and abnormal metabolic pathways, while PG2 and PG3 showed activation of cell damage signaling and immune-related pathways with reversed activation patterns (Fig. S6).

**Fig. 5:**
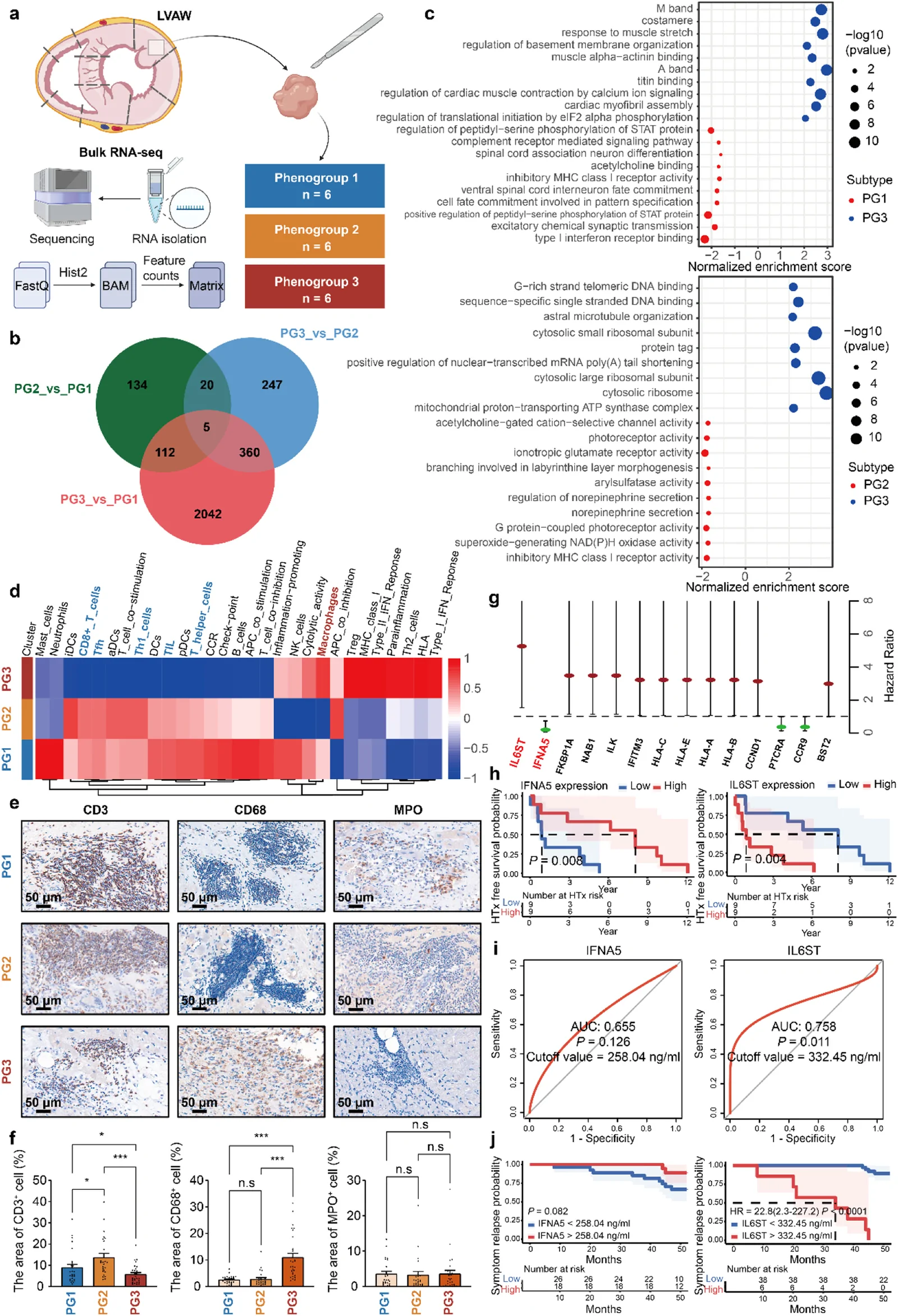
**Transcriptomic profiling and biomarker validation in aggressive myocarditis phenogroups.** (a) Overview for transcriptome analysis. (b) Venn diagram of differentially expressed genes (DEGs) from pairwise comparisons between pathological phenogroups (PG1, PG2, PG3). (c) GSEA enrichment analysis of DEGs for PG3 vs PG1 (top) and PG3 vs PG2 (bottom). (d) The ssGSEA immunoinfiltration heatmap derived from the transcriptome data of different PGs. (e) Representative immunohistochemical staining for CD3 (T lymphocytes), CD68 (macrophages), and MPO (neutrophils) in myocardial tissues. (f) Quantitative histomorphometry of IHC staining intensity (*P < 0.05, ^⁎⁎⁎^*P* < 0.001). (g) Forest plot of hazard ratios (HR) for 14 immune-related genes significantly associated with heart transplantation-free survival (*P* < 0.05), stratified by median gene expression. (h) K-M curves for IL6ST (highest HR) and IFNA5 (lowest HR). (i) ROC analysis of serum IL6ST and IFNA5 protein levels in an independent validation cohort. (j) Based on the optimal threshold obtained from ELISA (IL6ST/IFNA5), a KM survival analysis was conducted to examine the recurrence of symptoms.

The ssGSEA immunoinfiltration analysis revealed significant inflammatory characteristics among different PGs (Fig. 5d). PG1 and PG2 showed significantly enhanced T-cell-mediated immune responses, with an increased infiltration score of CD8⁺ T cells, as well as Th1 cells and helper T cells. These groups also exhibited enhanced activity in T-cell co-stimulation, cytolytic activity, and immune checkpoint pathways. In contrast, PG3 showed mainly innate immune activation, with a significant increase in macrophage infiltration, accompanied by enrichment of inflammatory promotion pathways, antigen-presenting cell (APC) co-stimulation, and type I/II interferon responses.

According to the literature, the immunobiological detection of inflammation is prognostically relevant. Here, different cell types including T cells, macrophages and neutrophils were characterized by immunohistology among the three PGs [8] (Fig. 5e–f). We found that macrophage infiltration was the feature of PG3, while T cell infiltration was the characteristic of PG2. There was more T cell infiltration than macrophage or neutrophil infiltration in PG1. The results were consistent with the results of immunoinfiltration analysis based on RNA-seq data (Fig. 5d).

### 3.7 Identifying IL6ST as a novel prognostic biomarker for myocarditis

In clinical practice, there was no effective predictive biomarker for the prognosis of aggressive myocarditis [33]. To identify biomarkers with clinical applicability, we conducted a systematic survival screening of 574 immune cell marker gene sets utilized in the ssGSEA-based immunoinfiltration analysis (Fig. 5d). Patients were stratified based on the median expression value of the transcriptome for each gene, and the risk of transplantation progression was subsequently evaluated in batches. Fourteen gene sets exhibited statistically significant prognostic value (*p* < 0.05). Among these, eleven genes were linked to poor prognosis, with IL6ST demonstrating the highest HR. Conversely, three genes were associated with a favorable prognosis, with IFNA5 showing the lowest HR (Fig. 5g). IFNA5 is an interferon-related gene, whereas IL6ST has been reported to be associated with the inhibition of gene function related to myocardial regeneration [34]. The corresponding K-M curves for transplantation progression are depicted in Fig. 5h

We validated these findings in an independent cohort of 45 clinically diagnosed myocarditis patients with an average follow-up duration of 46.3 ± 8.9 months. Plasma IL6ST levels > 332.45 ng/ml effectively distinguished patients with symptom recurrence (AUC = 0.758, *p* = 0.011; Fig. 5i), and the recurrence progression was notably faster (HR = 22.8, 95% CI 2.3 - 227.2; *p* < 0.0001; Fig. 5j). Although plasma IFNA5 levels exhibited a consistent directional trend, they did not achieve statistical significance (Fig. 5i–j). These findings suggest that IL6ST can serve as a clinically relevant biomarker for evaluating risk stratification in myocarditis.

### 3.8 Clinical promotion of pathological classification and supervised decision tree modelling to enhance clinical utility

To evaluate the clinical relevance of the pathological phenogroups, we conducted comprehensive correlation analyses that integrated clinical, genomic, and transcriptomic features with deep-learning classifications (Fig. 6). PG1 demonstrated strong associations with desmosome sarcomere-related gene mutations (e.g., TTN), early disease onset, and a familial history of cardiomyopathy, establishing its identity as the structural damage subtype. PG2 was characterized as the autoimmune subtype, based on dominant T-cell infiltration (immunohistochemistry: CD3^+^ cells; ssGSEA: Th1/TIL signatures) and the enrichment of myocarditis-related mutations. PG3 was defined as the virus infection-driven subtype due to its unique correlations with proviral infection and ventricular arrhythmias, further supported by macrophage-dominated inflammation (immunohistochemistry: CD68^+^ cells; ssGSEA: macrophage signature).

**Fig. 6:**
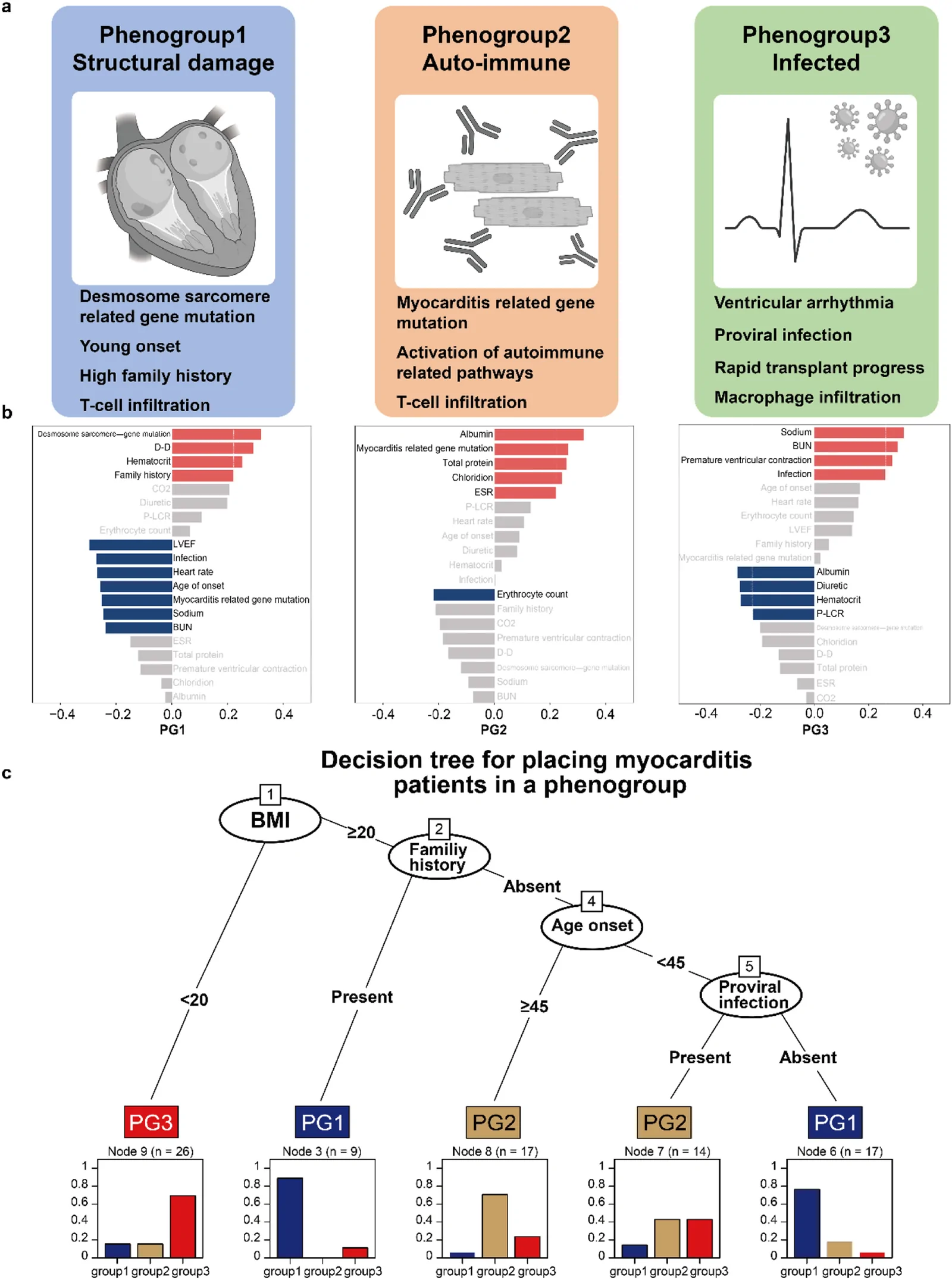
**Clinical significance of phenotypic grouping and construction of decision tree model.** (a) Naming of clinical features of the three groups of pathological phenotypes; (b) The coefficient ranking of the three pathological groups and their related clinical features; (c) Decision tree model construction.

Furthermore, to facilitate clinical application, we employed supervised decision tree modeling using the phenogroups from the clustering as input (Methods). The classifier was created using only a few clinical variables that differed among the three phenogroups. This classifier could effectively categorize patients into their corresponding phenogroups. A low number of variables in such a classifier enhances its potential applicability in clinical practice. Among the various clinical parameters across the three phenogroups, body mass index (BMI), family history presence, age at onset of myocarditis, and history of proviral infection were identified as the most significant variable characteristics, yielding a combined accuracy of 70% (Fig. 6c and Table S6). Additionally, we developed a decision tree classification software in Python. By inputting the aforementioned four parameters, the corresponding PG classifications can be output (Fig. S7).

## 4 Discussion

In this study, 83 heart transplant patients with aggressive myocarditis were reviewed retrospectively over the past 10 years at the Fuwai Hospital. Utilizing heart transplant samples of aggressive myocarditis patients, accurate pathology classification was performed. Each of these phenogroups had distinct clinical characteristics, gene mutation profiles and cardiac transcriptomic profiles. Then, a classifier was built to classify aggressive myocarditis patients quickly based on the four clinical data (BMI, presence of family history, age at onset of myocarditis, and proviral infection history). This classification was the first to integrate clinical features, cardiac function, comorbidities, and underlying etiology, which could pave the way for more targeted and personalized treatment approaches (Graphical abstract).

Given the challenging nature of diagnosing myocarditis in clinical practice, the lack of a pathological diagnosis often leads to a clinical suspicion of myocarditis. Endocardial biopsy remains the gold standard for clinical diagnosis, but it carries the risk of false negatives and may fail to pinpoint the lesion site, placing a psychological burden on the patient. A six-site pathology analysis of aggressive myocarditis transplant patients involved in our study fills this gap, providing accurate pathology insights based on actual myocarditis cases. This scheme proved invaluable in clinically diagnosing nearly half of suspected myocarditis patients, enabling us to stratify patients based on this protocol.

The classification of aggressive myocarditis into three phenogroups was based on a detailed pathological analysis (Fig.1). PG1, characterized by slow transplantation progress, exhibits mutations primarily in cardiac structural proteins, which overlap with mutations seen in dilated cardiomyopathy (DCM) and arrhythmogenic cardiomyopathy (ACM). This aligns with previous studies suggesting that patients in this subgroup have a compromised heart structure, resulting in chronic inflammation and slower progression compared to DCM and ACM. Transcriptomic analysis revealed damaged cardiomyocytes and abnormal energy metabolism in these patients. Clinically, a definite family history, poor heart function pre-transplant, and earlier age of onset are prominent features. Identifying this subgroup early is crucial, as distinguishing them from cardiomyopathy is essential for proper management. While patients with altered heart function and genetic mutations may be misclassified as having cardiomyopathy, inflammation plays a key role in early detection in this group. Anti-inflammatory therapy, in addition to management of heart failure, may slow down the progression of heart transplantation in these patients (PG1). According to the immunophenotyping (Fig. 5e–f), we found that PG1 and PG2 were characteristic of the T cell infiltration, which belongs to lymphocytic myocarditis. It is well known that immunosuppression is not routinely recommended for all forms of acute lymphocytic myocarditis. Recommendations for this and other forms of acute, complicated myocarditis are not supported by evidence from randomized clinical trials but are derived from case series and pathophysiological considerations. Therefore, more randomized clinical trials should be performed to support the use of immunosuppressive or immunomodulatory agents for PG1 and PG2. The fastest progress in transplantation among our phenogroups is seen in PG3. This group of patients with an elevated transplant risk showed significant pathological features in the inflammatory regions of the heart, including giant cell masses. Clinically, they have a history of proviral infection and a high incidence of ventricular arrhythmias. Transcriptome analysis revealed myocardial cell damage, abnormal immune activation, and multiple immune activation pathways associated with infection. This was in contrast to PG2 patients, who show a preference for autoimmune activation pathways on the transcriptome. PG2 and PG3 also exhibit more genetic mutations associated with immunity at the genome level than PG1, suggesting a genetic predisposition.

To facilitate clinicians in identifying patients with aggressive myocarditis, we developed a decision tree consisting of three pathological subtypes based on clinical characteristics (Fig. 6c). Additionally, we created a user-friendly program that aligns with the decision tree, with detailed instructions available (Fig. S7). Once a clinical patient arrives at the hospital, the decision tree can be employed if they exhibit signs suggestive of myocarditis following various evaluations such as physical examination, ultrasound, MRI, ECG, and primary complaint, and an endocardial biopsy is not feasible. By taking into account a patient's BMI, family history, age at diagnosis, and pre-infection history, our classification accuracy exceeds 70%, with a notable accuracy of over 80% for excluding PG1. Consequently, the findings from this study can help guide treatment decisions, as there is a lack of specific therapeutic strategies for different phenogroups. However, a “one-size-fits-all” approach, such as generalized anti-inflammatory or anti-aging therapies, may not be sufficient to delay the need for transplantation due to the varying inflammatory etiologies. Therefore, a thorough understanding of patient grouping, etiology, endocardial biopsy diagnosis, and tailored treatment appears to be essential.

Differences across the novel grouping were evaluated to identify which clinical or biomarker features were most important for the outcomes of aggressive myocarditis, to monitor the conditions in patients outside the hospital. While other biomarkers and cytokines, such as high-sensitivity cardiac troponin T and creatine kinase (CK), were used to diagnose myocarditis, they could not be used to predict a patient's prognosis. In our study, the interleukin-6 signal transducer (IL6ST), also known as gp130, was found to be the most predictive feature for patient outcomes (Fig. 5g–j). IL6ST is a transmembrane receptor for IL-6 and OSM. IL-6 and OSM are associated with both innate and adaptive immune cells and are associated with heart failure. IL-6 is a central inflammatory mediator and potential therapeutic target in heart failure [35], and IL-6/IL6ST signaling in CD4^+^ cells plays a critical role in the pathogenesis of pulmonary arterial hypertension [36]. In myocarditis, chronic IL-6 signaling has been reported to contribute to the progression to dilated cardiomyopathy [37]. IL6ST expression for PG3 was the highest comparing with other phenogroups, suggesting that it may be a marker of poor survival. The role of IL-6 in the development of cardiovascular disease is complex, and it has shown to have both beneficial and adverse effects in preclinical studies. However, it has not previously been associated with outcomes for aggressive myocarditis.

This study has some limitations. First, the sample size is relatively small and lack of virological data. Given our aim to classify aggressive myocarditis to guide treatment, expanding the sample size is challenging due to the limited number of transplantations worldwide [38]. In addition, our study was retrospective and we were not able to obtain sufficient cardiac tissue samples for the detection of a viral genome such that the presence of a prior viral infection could be assessed using the patient's past medical history. Second, we did not involve an external validation cohort, which makes replication on a global scale difficult. Additionally, the patients enrolled in our study all had aggressive myocarditis, and therefore, may not be representative of all stages of myocarditis cases. According to single-cell RNA-sequencing studies of viral myocarditis [39] and autoimmune myocarditis [40], pathology may be different in different stage of myocarditis. Recently, Professor Wang and his colleagues reported the largest cohort of histochemical analyses of fulminant myocarditis, based on endomyocardial biopsy samples, who were in early stage of myocarditis [41]. This study provides an overview of the clinicopathologic features of fulminant myocarditis. It was very meaningful to combine the different stages of myocarditis to systematically study the clinicopathological features of myocarditis. Lastly, clinical phenotypes of all the patients are based on traditional treatments, which are not effective. Through years of efforts of Chinese clinical scientists, Chinese guidelines provided unique treatments which reduced in-hospital mortality from over 50% to ∼5% of adult fulminant myocarditis [42]. A life support-based comprehensive treatment and immunoglobin have been reported to lower the in-hospital mortality of patients with fulminant myocarditis [43,44]. Whether these unique treatment modalities are effective for aggressive myocarditis requires further investigation. Despite these limitations, our pathological classification of aggressive myocarditis offers significant contributions to research in this area. Examining the entire transplant cohort underscores the importance of validating the various etiologies of aggressive myocarditis to better understand its progression to heart failure. This underscores the significance of customizing precise treatments for specific etiologies to delay the need for transplantation.

## 5 Conclusion

This study identified three distinct phenogroups of aggressive myocarditis, which showed significant differences in pathological manifestations, immunophenotyping, survival outcomes, clinical presentations, and molecular profiles. This finding improves patient selection for novel interventions and, ultimately, enables precision medicine.

## Data availability

The data, analytical methods, and study materials will be made available to other researchers for the purposes of reproducing the results or replicating the procedures. The matrix file of bulk RNA-seq, the MAF file containing mutation information filtered by whole-exome sequencing, as well as the classifier software and its source code have all been uploaded to GitHub (https://github.com/HanMofuwai/Data-myocarditis-classification). Additional data can be requested by contacting the corresponding author, Jiangping Song (fwsongjiangping@126.com).

## CRediT authorship contribution statement

**Xiumeng Hua:** Writing – review & editing, Writing – original draft, Funding acquisition. **Jiayu Zhang:** Methodology, Data curation, Conceptualization. **Han Mo:** Writing – review & editing, Validation, Data curation. **Xiao Chen:** Investigation. **Mengni Bao:** Supervision, Project administration. **Zhe Sun:** Validation, Project administration, Formal analysis. **Fei Dong:** Supervision, Investigation. **Yijing Li:** Visualization, Validation, Software. **Shun Liu:** Visualization, Supervision, Resources, Formal analysis. **Yiqi Zhao:** Formal analysis. **Weiteng Wang:** Methodology. **Zeyu Cui:** Resources. **Qian Zhao:** Resources. **Ningning Zhang:** Validation. **Jing Zhang:** Methodology. **Xiyue Wang:** Writing – review & editing, Writing – original draft, Methodology. **Jiangping Song:** Writing – review & editing, Writing – original draft, Funding acquisition.

## Declaration of competing interest

The authors declare that they have no conflicts of interest in this work.

## Appendix Supplementary materials

Image, application 1
