# Supplementary material for: Accurate pathologic classification of aggressive myocarditis improves insights into patient precise medicine
Source: Fundam Res. 2025 Sep 18;6(5):3431–44. doi: 10.1016/j.fmre.2025.09.008 (PMC13644499; doi:10.1016/j.fmre.2025.09.008)
Supplement: Supplementary file 1 [file mmc1.docx]

 **Fig. S1. KM survival curves of three pathologic phenotypic groups for transcriptome sequencing.**

**Fig S2. Transcriptomic comparisons across three pathological groups.** (a-c) Transcriptome difference analysis of three pathological groups volcano map. (d) Venn diagrams of the up-regulated and down-regulated genes that were differentially expressed among the three pathological groups were respectively presented.

**Fig S3. GO enrichment analysis of down regulated genes.** (a) only down regulated genes in PG2 vs PG1; (b) only down regulated genes in PG3 vs PG2; (c) only down regulated genes in PG3 vs PG1.

**Fig. S4. GO enrichment analysis of up regulated genes.** (a) only up regulated genes in PG2 vs PG1; (b) only up regulated genes in PG3 vs PG2; (c) only up regulated genes in PG3 vs PG1.

**Fig S5. GSEA analysis.** (a) the GO and KEGG enrichment analysis of GSEA under PG2 was compared in pathologic group PG3; (b) the GO and KEGG enrichment analysis of GSEA under PG1 was compared with pathologic group PG2; (c) GO and KEGG enrichment analysis of GSEA under PG1 was compared in pathologic group PG3.

**Fig S6. GSVA enrichment analysis of different pathologic phenotypes.**

**Fig. S7.** **Interactive clinical classifier for aggressive myocarditis phenogroups.** This interactive decision tree model enables non-invasive classification of aggressive myocarditis into three pathological phenogroups using four clinical inputs: Body Mass Index (BMI), age of disease onset, presence/absence of family history, and presence/absence of proviral infection history.
